# Rapid and Gentle Immunopurification of Brain Synaptic Vesicles

**DOI:** 10.1523/JNEUROSCI.2521-21.2022

**Published:** 2022-04-27

**Authors:** Mazdak M. Bradberry, Shweta Mishra, Zhao Zhang, Lanxi Wu, Justin M. McKetney, Martha M. Vestling, Joshua J. Coon, Edwin R. Chapman

**Affiliations:** ^1^Department of Neuroscience and Howard Hughes Medical Institute, University of Wisconsin School of Medicine and Public Health, Madison, Wisconsin 53705; ^2^Medical Scientist Training Program, University of Wisconsin School of Medicine and Public Health, Madison, Wisconsin 53705; ^3^Department of Chemistry, University of Wisconsin–Madison, Madison, Wisconsin 53706; ^4^Department of Biomolecular Chemistry and Genome Center of Wisconsin, University of Wisconsin–Madison, Madison, Wisconsin 53706; ^5^Morgridge Institute for Research, Madison, Wisconsin 53715

**Keywords:** biochemistry, cryo-electron microscopy, lipid metabolism, neurodegeneration, proteomics, synaptic vesicles

## Abstract

Current methods to isolate synaptic vesicles (SVs), the organellar quanta of synaptic transmission, require highly specialized materials and up to 24 h. These technical obstacles have thus far limited the study of SVs in models of synaptic function and pathophysiology. Here, we describe techniques for the rapid isolation of SVs by immunoprecipitation with widely available antibodies conjugated to magnetic beads. We report that the inexpensive rho1D4 monoclonal antibody binds SVs and show that elution with the 1D4 peptide yields native vesicles that are ≥ 10-fold purer than those obtained with classical techniques. These methods substantially widen the accessibility of SVs, enabling their purification in 60–90 min for downstream analyses including mass spectrometry and cryo-electron microscopy. Immunopurified SV preparations from mouse brain contained apolipoprotein E, the LDL receptor Lrp1, and enzymes involved in lipid metabolism, suggesting that SVs may play direct roles in lipid homeostasis and lipoprotein trafficking at the nerve terminal.

**SIGNIFICANCE STATEMENT** SVs are small organelles that form and recycle at nerve terminals to enable synaptic transmission. Much remains unknown about the processes that enable the formation and function of SVs. Moreover, nerve terminals appear to be particularly vulnerable to pathophysiologic processes underlying neurodegenerative diseases and schizophrenia. Although techniques to purify synaptic vesicles thus have the potential to yield significant insights into physiology and pathophysiology of nerve terminals, current methods rely on either esoteric materials or expression of transgenes. This article addresses these problems by establishing robust, efficient methods for SV purification using widely available materials, and it highlights several promising areas of future study arising from proteomic analyses of immunopurified SVs.

## Introduction

Synaptic vesicles (SVs) are small (∼40 nm diameter) organelles that store neurotransmitters within nerve terminals and rapidly undergo exocytosis upon presynaptic Ca^2+^ influx. The current standard for high-yield, high-purity SV purification for biochemical analysis relies on the size, physical properties, and abundance of these organelles in the brain (Ahmed et al., 2013). Based on techniques developed in the 1960s (Whittaker et al., 1964), this approach takes ∼24 h and involves gradient centrifugation and sedimentation of vesicles at high speed before size exclusion chromatography. The chromatography media typically used for the final size-exclusion step, controlled pore glass or Sephacryl S-1000, are no longer widely available, and these preparations are unavoidably contaminated with other cellular components such as glutamine synthetase (Ahmed et al., 2013; Taoufiq et al., 2020). Other approaches involving purification of SVs by immunoprecipitation (IP) have been limited by elution steps that damage or destroy the resin-bound vesicles (Burger et al., 1989; Takamori et al., 2000; Chantranupong et al., 2020). These techniques have been valuable in determining SV membrane composition (Takamori et al., 2006), neurotransmitter content (Burger et al., 1989, 1991; Chantranupong et al., 2020), and transport activity (Burger et al., 1989, 1991; Takamori et al., 2000). However, each of these approaches also requires highly specialized materials, such as large quantities of proprietary antibodies or the expression of exogenous genes, and may yield vesicles of reduced purity compared with classical methods (Chantranupong et al., 2020). Investigators seeking deeper insights into the biogenesis, composition, physical properties, function, and pathophysiological relevance of SVs would be substantially aided by gentler, more selective, and more convenient procedures for their isolation.

This article describes advances to immunoprecipitation-based approaches for SV purification that substantially reduce the barrier to entry for obtaining pure SVs. We demonstrate the suitability of these approaches for analysis of SVs by cryo-electron tomography and liquid chromatography-mass spectrometry (LC-MS). Our LC-MS proteomics results provide unequivocal evidence of the purity of these preparations and suggest previously unappreciated roles for SVs in lipid metabolism and lipoprotein trafficking.

## Materials and Methods

### Animals

C57B/6J mice of either sex between 14 and 20 d of age were used for all experiments. All work was conducted according to protocols approved by the University of Wisconsin Institutional Animal Care and Use Committee.

### Antibodies for immunoprecipitation

Mouse monoclonal anti-syt1 (synaptotagmin-1) antibody (mAb 48; Matthew et al., 1981) and anti-SV2 (Buckley and Kelly, 1985) antibodies were purified by protein G chromatography from stocks of ascites generated before the year 2010. Rho1D4 mAb was purchased from the University of British Columbia (https://ubc.flintbox.com). Indistinguishable results were obtained using two batches of rho1D4 mAb manufactured in 2017 and 2020. Bovine IgG was purchased from Sigma-Aldrich. For most experiments, all antibodies were dialyzed extensively against PBS (140 mm NaCl, 10 mm sodium phosphate buffer, pH 7.4) before bead coupling, but indistinguishable results were also obtained using rho1D4 directly as provided by the manufacturer in PBS. All antibodies were aliquoted and stored frozen at −20°C before bead coupling. We observed loss of activity in rho1D4 antibody aliquots that underwent evaporation during storage at −20°C and thus recommend that care be taken to ensure this does not occur, for example, by storing at lower temperatures.

### Bead preparation

Dynabeads M-270 epoxy (300 mg, catalog #14302D, Thermo Fisher Scientific) were stored at 30 mg/ml in anhydrous, amine-free N,N-dimethylformamide (DMF) for up to 18 months before coupling. Nonautoclaved tubes were used for all steps involving Dynabeads. For each coupling reaction, 10 mg beads were transferred to a fresh 1.7 ml polypropylene microcentrifuge tube, and the DMF was removed after collecting the beads with a magnetic stand (Promega). A 250 μg aliquot of each antibody was brought up to 200 μl with borate buffer (100 mm sodium borate, pH 8.5), and this was used to thoroughly resuspend the beads. An additional 200 μl borate buffer was added to each tube and mixed by pipetting up and down, followed by the addition of 200 μl 3 m ammonium sulfate in borate buffer and further mixing by pipetting up and down. The reaction mixture was incubated with rotation at 37°C overnight. After removal of the supernatant, the beads were washed by thorough resuspension with three cycles of 1 ml 500 mm NaCl and 50 mm ammonium acetate, pH 4.5, followed by 500 mm NaCl and 50 mm Tris-HCl, pH 8.0 (6 × 1 ml washes total, alternating between Tris and ammonium acetate solutions). The Ab-Dynabeads beads were then washed (2 × 1 ml) and resuspended at 30 mg/ml in 150 mm KCl and 50 mm Tris-HCl, pH 8.0, and stored at 0–4°C until use. For some experiments, 145 mm KCl and 10 mm potassium phosphate buffer, pH 7.2, were used as the final wash and storage buffer, with no discernable change in performance. Preliminary experiments using alternative coupling strategies, including N-hydroxy-succinimidyl ester activation of Dynabeads carboxylic acid and chemical cross-linking to Dynabeads Protein G, gave poorer yields and unacceptably high antibody contamination in the eluates.

### SV immunoisolation

**Figure 1.**
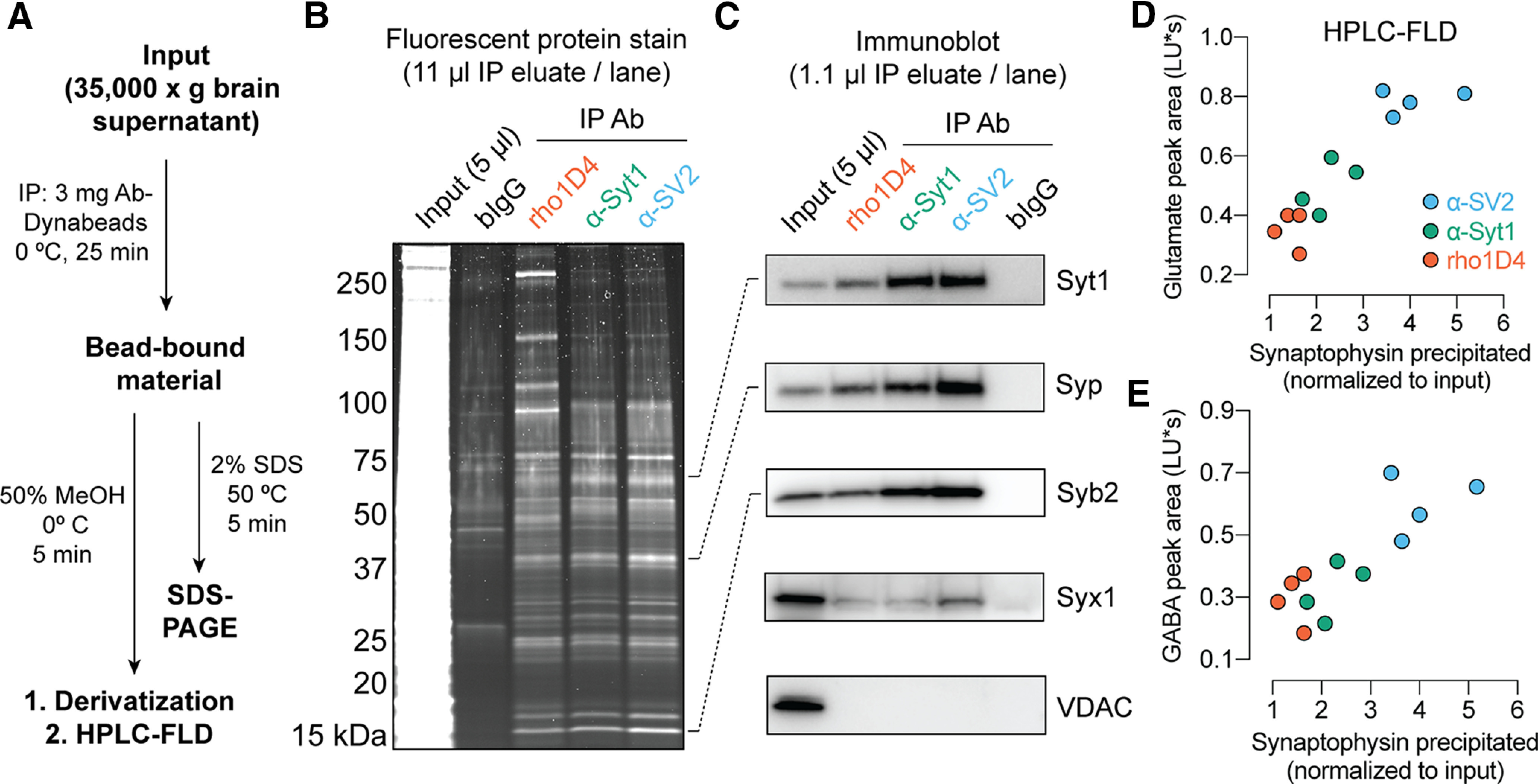
Immunopurification of synaptic vesicles. ***A***, Scheme for vesicle immunoprecipitation and analysis. Each mouse brain provided sufficient material for analysis of protein and neurotransmitter using two different mAbs. ***B***, Staining of proteins separated by SDS-PAGE demonstrates broad similarity among anti-SV2, anti-syt1, and rho1D4 immunoprecipitates, with minimal protein binding by control beads bearing pooled bovine IgG. Note the dominant band at 38 kDa, corresponding to synaptophysin. ***C***, Immunoblot analysis of precipitated material. Each antibody yields strong enrichment of SV proteins, but only the expected weak enrichment of the plasma membrane t-SNARE syntaxin-1 and no detectable contamination from the mitochondrial protein VDAC. ***D***, ***E***, For each experiment, the area of the HPLC fluorescence peak corresponding to NBD-derivatized glutamate (***D***) or GABA (***E***) was plotted against the normalized intensity of the synaptophysin band on immunoblot (*n* = 4 biological replicates using two separately prepared batches of Ab-Dynabeads for each mAb; ***B***–***E***). Example raw chromatograms used for GABA and glutamate measurements are shown in Extended Data Figure 1-1. “LU*s”, luminance units * seconds.

All buffers and equipment were cooled to 0–1°C before beginning experiments, and all operations from homogenization until bead elution were conducted in a cold room. One to two C57B6/J mice, postnatal day (P) 14-20, were killed, and the brains, including cerebellum and brainstem, were rapidly removed. Each brain was homogenized in 4.2 ml of homogenization buffer [125 mm KCl, 20 mm potassium phosphate buffer, 5 mm EGTA, and protease inhibitors (cOmplete Mini EDTA-free, 1 tablet/10 ml), pH 7.3, at 0°C], using 10 strokes in a Teflon-glass Dounce homogenizer with rotation at 850–900 rpm using a digital overhead mixer (IKA). The homogenate was then centrifuged (20 min, 35,000 × *g*, 1°C). During centrifugation, 3 mg of Ab-Dynabeads (∼100 μl slurry) were transferred to a fresh 2 ml microcentrifuge tube, washed with 1 × 1 ml wash buffer (150 mm KCl, 10 mm potassium phosphate buffer, pH 7.2, at 0°C), and resuspended with 100 μl homogenization buffer. Following centrifugation, the supernatants (∼2.5 mg/ml protein) were pooled, and 1.9 ml was added to each tube containing Ab-Dynabeads. The tubes were incubated with rotation for 25 min, with the temperature maintained at 0°C by placing the tubes inside 50 ml conical tubes packed with ice. The beads were then collected using a magnetic stand, the supernatant was discarded, and the beads were washed four times by gently resuspending and triturating in 1 ml ice-cold wash buffer. In all cases, the final wash was used to transfer the beads to fresh 1.7 ml microcentrifuge tubes for elution. For Figure 1, the beads were split into two equal portions (1.5 mg each). For protein analysis, one portion was eluted by adding 30 μl 2% SDS and 25 mm Tris, pH 8.0, and heating to 50°C for 5 min. For polar amine analysis, the other portion was eluted by adding 30 μl of 50:50 MeOH:borate buffer and incubating on ice for 5 min. In Figure 1, immunoprecipitations using all four Ab-Dynabead conjugates were conducted in parallel for each experiment. For native elution experiments, the rho1D4 beads (3 mg; see Fig. 3) bearing SVs were washed as above and incubated first with 50 µl 200 μm 1D4 peptide (Cube Biotech) for 30 min on ice. This eluate was transferred to a fresh tube, and the beads were then eluted in 50 µl 2% SDS with heating to 50°C for 5 min. The elution buffers included 100 mm sodium borate, pH 8.5 (see Fig. 3) or 140 mM NaCl and 25 mM HEPES-NaOH, pH 7.4 (see Fig. 4). SVs were readily eluted with the 1D4 peptide in all buffers tested, for example, containing the following (in mm): 135 NaCl; 25 HEPES-NaOH, pH 7.4; 5 EGTA; 100 KCl; 25 HEPES-NaOH, pH 7.4; and 200 ammonium acetate (see Fig. 4). In later experiments (data not shown), higher concentrations of peptide (∼1 mm) appeared more effective for eluting SVs, and so we encourage investigators using this method to titrate this peptide elution step during initial studies.

### SDS-PAGE and immunoblot

Following elution with peptide or SDS, one-third volume of 4× SDS sample buffer containing β-mercaptoethanol or dithiothreitol (DTT) was added to the eluate, and the input fraction was prepared by mixing 30 μl brain supernatant with 60 μl 4× SDS sample buffer. Samples were reduced and denatured by heating to 50°C for 15 min and subjected to SDS-PAGE on 4–20% polyacrylamide gradient gels (TGX, Bio-Rad). For protein analysis, 15 μl of each sample was loaded, and the gel was fixed in MeOH-acetic acid and stained with Lumitein One-Step fluorescent protein stain (Biotium; Fig. 1, see Fig. 3). For immunoblots as shown in Figure 1, the same amount of input was run, but the immunoprecipitation samples were diluted 10-fold in sample buffer before SDS-PAGE. Proteins were transferred to PVDF membranes, blocked in 5% nonfat dry milk in 150 mm NaCl, 10 mm Tris, pH 7.4, plus 0.1% Tween 20 (TBS-T), and incubated overnight with primary antibody in TBS-T containing 1% nonfat dry milk. The primary antibodies used were the following: mouse anti-synaptobrevin monoclonal (clone 69.1, 1:1000 dilution of an 0.1 mg/ml stock; catalog #104 211, Synaptic Systems), guinea pig anti-synaptophysin polyclonal (1:1000 dilution of a 0.5 mg/ml stock; catalog #101 004, Synaptic Systems), mouse anti-synaptotagmin monoclonal (mAb 48, 1:1000 dilution of a 3.7 mg/ml stock purified from ascites fluid; Developmental Studies Hybridoma Bank), mouse anti-syntaxin monoclonal (clone HPC-1, 1:1000 dilution of a 3 mg/ml stock; catalog #ab3265, Abcam), and rabbit polyclonal anti-VDAC (voltage-dependent anion channel; 1:1000 dilution of the supplied solution; catalog #AB10527, Millipore). HRP-labeled secondary antibodies were used for detection. Synaptophysin band intensity was determined in ImageJ using blank adjacent lanes for background subtraction.

### Quantification of SV protein enrichment

Input protein concentration was determined by the BCA assay (Pierce). Because peptides and detergents interfere with most protein assays, concentration of rho1D4-IP eluates was estimated on fluorescently stained SDS-PAGE gels using dilutions of BCA-quantified input material as a calibration curve. The amount of synaptophysin in rho1D4-IP eluates and input was determined by immunoblot, with dilutions performed to achieve similar band intensities for each fraction. Enrichment was calculated as the ratio of synaptophysin band intensity to total protein concentration, which was normalized to a value of 1 for the input supernatant. Values for rho1D4 bead-bound material were calculated by summing contents of the peptide- and SDS-eluted material from each batch of beads.

### Amine analysis

For experiments in Figure 1, 8 μl of bead eluate in 50:50 MeOH:borate buffer was combined with 4 μl of freshly prepared derivatization solution comprising 5 mg/ml NBD-Cl (4-chloro-7-nitro-1,2,3-benzoxadiazole) in MeOH and heated to 60° for 70 min on a PCR block. 7-nitro-1,2,3-benzoxiadiazole (NBD) derivatized amines were analyzed on an HPLC system (Agilent Infinity 1260 Bio-Inert) with a reversed-phase C18 column (Agilent Peptide Mapping, 2.1 × 150 mm, 2.7 μm particle size) and fluorescence detector (Agilent 1260 FLD Spectra, 470 nm excitation, 530 nm emission). The instrument was operated using a manual injector, the column was kept at 40°C, and samples were applied to overfill a 5 µl sample loop. Mobile phase A was 95:5 H_2_O:MeCN containing 10 mm ammonium acetate, and mobile phase B was 95:5 MeCN:H_2_O containing 5 mm ammonium acetate. For Figure 1, GABA and glutamate were separated using an optimized gradient as follows: 0–20 min, 10−100% B; 20–25 min, 100% B; 25–30 min, 100−10% B. Peak identities were confirmed by running authentic standards, and areas were determined by integration in Agilent Chemstation software.

### Dynamic light scattering and negative-stain transmission electron microscopy

Three microliters of rho1D4 peptide eluate were analyzed in a micro cuvette on a dynamic light-scattering instrument (Wyatt). For each sample, 10 acquisitions were performed, and data from those acquisitions showing good autocorrelation functions (>90% of scans) were averaged to produce a single replicate. Data corresponding to the distribution of particle diameters were exported and plotted in Prism. Transmission electron microscopy (TEM) samples were prepared by uranyl formate staining of peptide-eluted SVs on glow-discharged grids.

### Cryo-EM sample preparation and analysis

Rho1D4 beads (3 mg) bearing SVs from one-half mouse brain were eluted in 50 µl of HEPES elution buffer containing the following (in mm): 25 HEPES, 140 NaCl, and 200 1D4 peptide. For cryoEM, 3 µl of the purified SV suspension was adsorbed onto 2 nm carbon-layer-coated Quantifoil R1.2/1.3300 mesh copper grids, glow discharged for 15 s using GloQube glow discharge system, and blotted for 2 s at 4°C and 95% humidity using a Vitrobot Mark IV (Thermo Fisher Scientific). Ten nanometer gold fiducial particles were used for tilt-series alignment. All images were acquired on a Talos Arctica 200 kV TEM using a Gatan K3 direct electron detector in correlated double-sampling counting mode, with an energy filter set to a 20 eV slit width. Nineteen tilt series were recorded at 31,000× magnification (2.89 Å/pixel), from −48 to 48° with 3° increments, at a dose rate of 15e^−^/Å/s and defocus of −3 µm. Tilt series alignment and tomographic reconstruction were done using the IMOD package. After preprocessing to remove x-ray artifacts and coarse alignment, a seed model was generated using 25 seed points. A fiducial model was generated by tracking individual seed points and filling the gaps. The fiducial model was then edited by moving the residuals to the center of the gold particles, if not already there. Sample tomogram thickness was set to 500 arbitrary units, and the tomogram was binned down by three to improve contrast. A boundary model was created with six contour lines enclosing the sample at three different *Y* locations in the tomogram to ensure the specimen is flat and centered along the *Z* axis. The final alignment stack was then generated, and the tomogram was computed. For each SV, diameters along the longest axis and the axis perpendicular to it were measured manually in IMOD (outer layer to outer layer of the lipid bilayer). To determine the longest axis, multiple axes at the largest cross section of the SV were measured, and the longest among them was chosen. The ratio of the diameters was used to calculate ellipticity of the SVs. Graphs were plotted using Prism, and 3D rendering of the SVs was done using IMOD.

### Sample preparation for LC-MS

**Figure 2.**
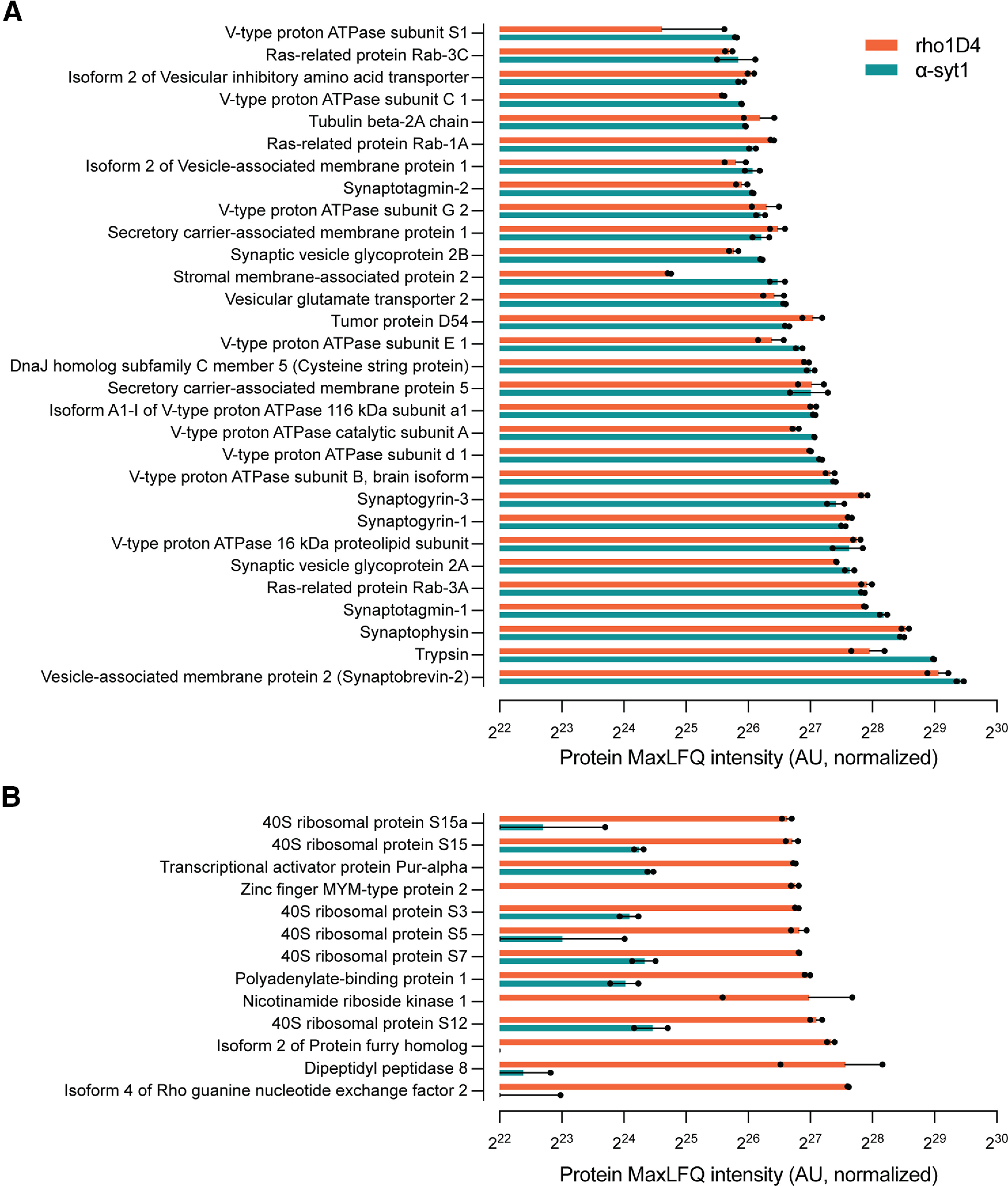
Proteomic characterization of α-syt1 and rho1D4 immunoprecipitated SVs. ***A***, Proteins identified by Q-TOF LC-MS after bead elution with SDS were sorted by intensity in the α-syt1 immunoprecipitates (*n* = 2 biological replicates per immunoprecipitation antibody). Intensity values were calculated using the IonQuant function and MaxLFQ algorithm in the FragPipe analysis software. The proteins with the top 30 highest intensity scores in α-syt1 IP samples, not including antibody fragments, are shown here. The majority of identified proteins are well known to reside on SVs and are well matched in intensity by the rho1D4-precipitated material, confirming that the rho1D4 mAb effectively immunoprecipitates a population of SVs similar to those obtained with the α-syt1 antibody. ***B***, Proteins among the top 30 in SDS-eluted rho1D4 IP samples that were not observed among the top 30 in α-syt1 IP samples. Source data used to generate this figure are shown in Extended Data Figure 2-1.

SVs from 0.5–1 mouse brain, eluted by treatment with SDS (2% w/v) or 1D4 peptide (200 μm), were prepared for liquid chromatography and mass spectrometry (LC-MS) using the SP3 method (Hughes et al., 2019). Ten percent w/v SDS was added to peptide-eluted samples to reach a final SDS concentration of 2% w/v. Reduction and denaturation was achieved by adding DTT (100 mm freshly prepared aqueous stock solution) to a final concentration of 5 mm and incubating at 50°C for 25 min. Cysteine alkylation was achieved by adding iodoacetamide (200 mm freshly prepared aqueous stock solution) to a final concentration of 15 mm and incubating at room temperature in the dark for 30 min, followed by quenching of iodoacetamide by further addition of DTT (26 mm DTT total). Dynabeads M-270 carboxylic acid (catalog #14305D, Thermo Fisher Scientific) were then added (2 µg/µl final concentration), and the tubes were mixed well, followed by the addition of 1 volume of absolute ethanol and brief incubation on a thermomixer (5 min, 1000 rpm, 23°C) to drive protein adsorption to the beads. The beads were washed three times with 200 µl 80% ethanol and transferred to a fresh tube with the final wash. Following removal of the supernatant, tryptic peptides were eluted from the beads by overnight digestion in 25–30 µl trypsin solution (0.01 µg/µl in 100 mm ammonium bicarbonate; catalog #V5111, Promega) with shaking in a thermomixer (1000 rpm, 37°C). Eluates from this step were used directly for LC-MS. The data presented in Figure 2 and Extended Data Figure 2-1 were obtained from experiments independent of those presented in Figure 5 and Extended Data Figure 5-1.

### LC-MS data acquisition

For Figure 2 and Extended Data Figure 2-1, data were acquired using a quadrupole time-of-flight mass spectrometer (Impact II, Bruker) connected to a nanoflow HPLC system (nanoACQUITY, Waters) via an electrospray ionization source. The HPLC was equipped with a nano-HPLC column [Waters ACQUITY UPLC Peptide BEH C18, 1.7 µm particles, 20 cm long, 75 µm inner diameter (i.d.)], preceded by a trap column (Waters ACQUITY UPLC M-Class Symmetry C18, 5 µm particles, 20 mm long, 180 µm i.d.), and held at 60°C. Mobile phase A was 0.2% formic acid in H_2_O, and mobile phase B was 0.2% formic acid in MeCN. The flow rate was 6 µl/min for trapping followed by 0.3 µl/min for elution, and the following gradient was used: 0–5 min, 5% B; 5–60 min, 5−55% B; 60–75 min, 55−90% B; 75–82 min, 90% B; 82–85 min, 90−5% B; 85–95 min, 5% B. MS^2^ data were acquired by fragmentation of the top 30 precursors for each MS^1^ survey scan. Precursor ions were dynamically excluded for 30 s after being detected in two spectra.

In a separate set of experiments, new samples were collected, and data were acquired using a hybrid Orbitrap mass spectrometer (Oritrap Fusion Eclipse, Thermo Fisher Scientific) connected via an electrospray ionization source (Nanospray Flex, Thermo Fisher Scientific) to a nanoflow HPLC system (Ultimate 3000 RSLCnano, Thermo Fisher Scientific; see Fig. 5, Extended Data Fig. 5-2). The HPLC was equipped with a capillary nano-HPLC column (PicoTip, SIS, 25 cm long, 75 µm i.d.) packed in house at ultra-high pressure (Shishkova et al., 2018) with 1.7 µm C18 particles (BEH C18, Waters) and held at 50°C using an in-house fabricated column heater. Mobile phase A was 0.1% formic acid in H_2_O, and mobile phase B was 0.1% formic acid in 80:20 MeCN:H_2_O. Injection volume was 3 µl. Peptides were separated in a 2 h gradient as follows: 0–17 min, 0−15% B; 17–102 min, 15–50% B; 102–104 min, 50−100% B; 104–108 min, 100% B; 108–110 min, 100−0% B. The flow rate was 300 nl/min, and the spray voltage was 2 kV. MS^1^ spectra were acquired in positive mode with the Orbitrap at 1 Hz with the following settings: resolution, 120,000; scan range, 400–1600 m/z; maximum injection time, 50 ms; AGC target, 400,000; normalized AGC target, 100%. MS^2^ spectra were likewise acquired in positive mode in the Orbitrap with the following settings: resolution, 30,000; scan range, 150–1800 m/z; maximum injection time, 60 ms; AGC target, 50,000; normalized AGC target, 100%; HCD collision energy, 30%. MS^1^ peaks were filtered based on the following criteria for fragmentation: charge state, 2–8; maximum intensity, 1E20, minimum intensity, 50,000. Monoisotopic precursor selection was used in peptide mode, and MS^1^ peaks were dynamically excluded for 20 s with a 20 ppm mass tolerance after being selected for fragmentation.

### LC-MS data analysis

Raw files were directly analyzed using the FragPipe data processing pipeline (Kong et al., 2017). Peptide spectral matching was performed using a mouse proteome database downloaded from Uniprot in June 2021 with the following settings: precursor mass tolerance, 50 ppm; fragment mass tolerance, 20 ppm; mass calibration and parameter optimization enabled; up to 2 missed cleavages; peptide length, 7–50; peptide mass range, 500–5000 Da. Cysteine carbamidomethylation was selected as a fixed modification and methionine oxidation and N-terminal acetylation as variable modifications. PeptideProphet and ProteinProphet were used for validation and assignment of peptide and protein results. Samples from each experiment were analyzed together using IonQuant (Yu et al., 2021) with match-between-runs (MBR) enabled and label-free quantification with normalization between experiments performed using the MaxLFQ algorithm with the following settings: feature detection m/z tolerance 10 ppm, feature detection retention time tolerance 0.4 min, MBR retention time (RT) tolerance, 1 min; MBR min correlation, 0; MBR ion false discovery rate (FDR), 0.01; MBR top runs 10; MBR peptide FDR 1; MBR protein FDR 1. Razor intensities for selected proteins were plotted in Prism (GraphPad). For comparison with data from Taoufiq et al. (2020), proteins detected in at least one experiment were included, and gene names were homologized using the SynGO ID convert tool (https://www.syngoportal.org/convert.html; Koopmans et al., 2019) before analysis of overlap using R.

### Data availability

The raw data (see Fig. 5) are freely available online via the Chorus project at https://chorusproject.org/anonymous/download/experiment/-8025126159725249575.

## Results

While developing techniques for SV immunopurification, we were surprised by preliminary findings that the rho1D4 mAb readily immunoprecipitated SV proteins and neurotransmitters from various mouse brain preparations. The rho1D4 mAb binds the C terminus of bovine rhodopsin (MacKenzie et al., 1984) and is presently available at a relatively low cost as a reagent for recombinant protein purification. Although this antibody would not be expected to bind SVs, our preliminary results motivated further testing of its suitability for SV immunoprecipitation. Pull-down experiments adapted from established procedures (Burger et al., 1991, 1989; Chen et al., 2016) were thus performed to compare the rho1D4 mAb to established mAbs against the ubiquitous SV proteins syt1 and SV2A/B/C (SV2; Fig. 1*A*). Antibody-bead conjugates were made using epoxy-coated 2.7 μm magnetic beads, which enabled direct covalent coupling of antibodies in a single overnight incubation step. After coupling to magnetic beads, each antibody precipitated a similar subset of the input proteome (Fig. 1*B*). For each immunoprecipitate, the protein bands observed on reducing SDS-PAGE corresponded reasonably well to those observed in previous studies of SVs isolated by either conventional means or immunoprecipitation (Jahn et al., 1985; Burger et al., 1989; Ahmed et al., 2013; Fig. 1*B*). In particular, a dominant band at 38 kDa, representing synaptophysin, was observed, along with strong bands at ∼65 kDa, representing syt1 and vesicular transporters, and 18 kDa, representing synaptobrevin (Fig. 1*C*). Among the antibodies used here, the anti-SV2 mAb precipitated SVs most efficiently, likely because of higher affinity for SVs versus the other mAbs. In each case, the bead-bound fraction was highly enriched in SV proteins and devoid of contaminants from mitochondrial membranes (Fig. 1*C*). The putative identities of major contaminants in the rho1D4 immunoprecipitates, which included four bands ∼90–150 and >250 kDa, were established by LC-MS (see below).

10.1523/JNEUROSCI.2521-21.2022.f1-1 Figure 1-1 Chromatograms of derivatized polar amines eluted from immunoprecipitated SVs. Amines were detected by reverse-phase HPLC with fluorescence detection following elution from beads with 50% methanol and derivatization with NBD chloride. The elution positions of GABA and glutamate were established using standards. In accordance with other metabolomic studies of SVs (Burger et al., 1991; Chantranupong et al., 2020), GABA and glutamate were the predominant amines detected in this sample. Download Figure 1-1, TIF file.

Fluorescence HPLC analysis of polar amines eluted from the beads demonstrated the predominance of glutamate and GABA, as expected for pure SVs isolated at 0°C (Fig. 1*D***–***E*; Extended Data Fig. 1-1; Burger et al., 1991; Chantranupong et al., 2020). These data likewise indicate the absence of intact lysosomes or mitochondria, which contain many other polar amines (Chen et al., 2016; Abu-Remaileh et al., 2017). Beads coated with pooled bovine IgG did not immunoprecipitate SV proteins (Fig. 1) or vesicular amino acid neurotransmitters (Extended Data Fig. 1-1). The amount of neurotransmitter immunoprecipitated by each antibody correlated well with the yield of synaptophysin, further suggesting that these antibodies pull down similar sets of vesicles despite differences in affinity for SVs (SV2 > syt1 > rho1D4; Fig. 1 *D–E*).

Proteomic analysis of syt1− and rho1D4 immunoprecipitates using trypsin digestion and LC-MS (Fig. 2, Extended Data Fig. 2-1) confirmed the purity of the immunoprecipitated material. In α-syt1 immunoprecipitates, well-known SV proteins made up nearly all the most abundant proteins detected (Fig. 2*A*, Extended Data Fig. 2-1). These proteins include those observed by Western blot (Fig. 1), vesicular transporters for GABA and glutamate, and a wide assortment of Rab GTPases and other proteins associated with SVs (Takamori et al., 2006; Taoufiq et al., 2020; Fig. 2*A*). Subunits of both the V_0_ and V_1_ sectors of the vesicular ATPase were among the most intense observed proteins, also in accordance with previous studies (Taoufiq et al., 2020). As expected from the protein bands observed by SDS-PAGE (Fig. 1), SV proteins were observed in similar intensity in the rho1D4 bead-bound material (Fig. 2*A*), again demonstrating that the rho1D4 mAb immunoprecipitates a population of vesicles similar to that obtained with antibodies against SV proteins. The primary presumed contaminants in the rho1D4 bead-bound material included the Rho guanine nucleotide exchange factor Arhgef2 (predicted mass ∼112 kDa), dipeptidyl peptidase 8 (Dpp8, 102 kDa), protein Furry homolog (Fry, 339 kDa), nicotinamide riboside kinase 1 (22.3 kDa), and zinc finger MYM-type protein 2 (Zmym2, 155 kDa), along with a number of ribosomal proteins (Fig. 2*B*). These observations correspond well to the protein banding pattern observed in SDS-PAGE for rho1D4 immunoprecipitates (Fig. 1).

10.1523/JNEUROSCI.2521-21.2022.f2-1 Figure 2-1 This table contains the results of two independent mass spectrometry experiments processed via FragPipe and described in Figure 2. This table accompanies this article as a Microsoft Excel file with further instructions for its use noted on the first sheet. Download Figure 2-1, XLSX file.

**Figure 3.**
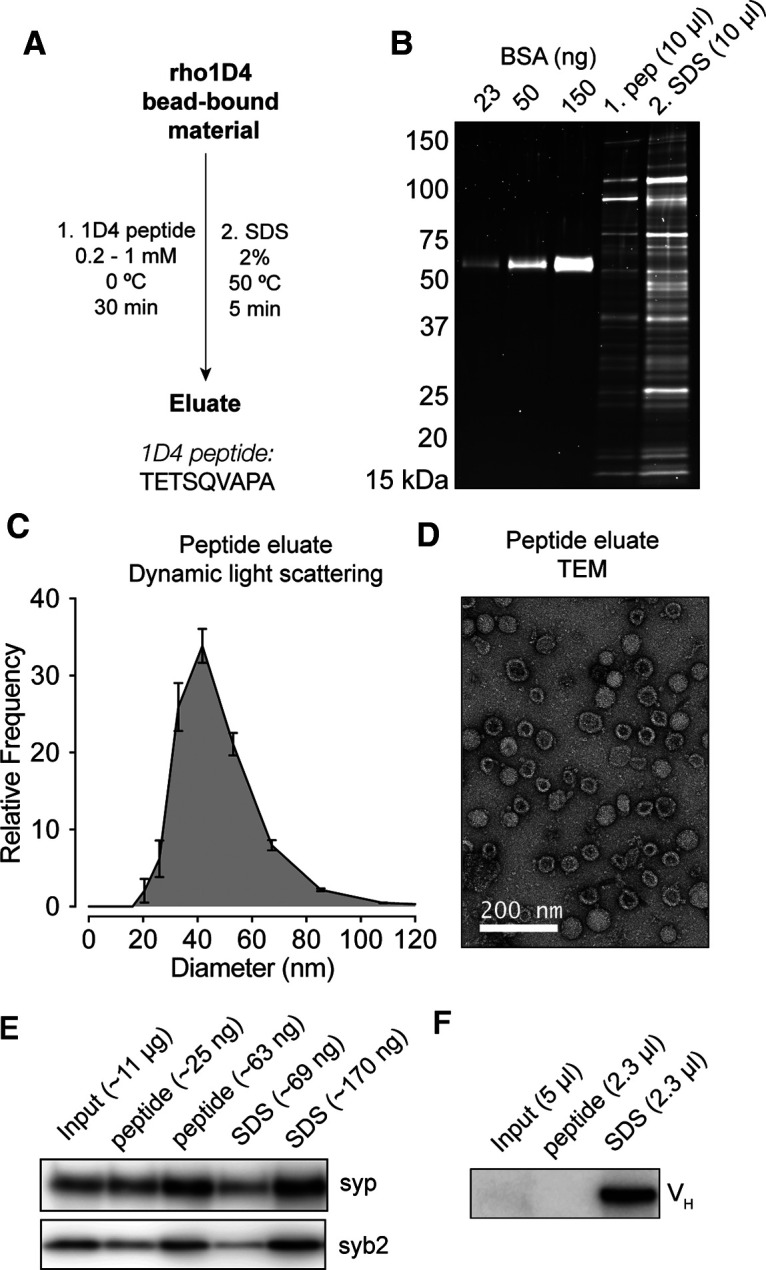
rho1D4-IP enables peptide elution of native SVs. ***A***, Purification scheme for peptide elution. The amino acid sequence of the 1D4 peptide sequence is shown. ***B***, SDS-PAGE with fluorescent stain of protein eluted from rho1D4 beads using the 1D4 peptide followed by 2% SDS. SV proteins were readily eluted from the beads with the 1D4 peptide. ***C***, Dynamic light scattering measurements (*n* = 3 biological replicates) of eluted material indicates a single population of particles ∼40–50 nm in diameter. This population represented >99% of particles detected in each sample. ***D***, Negative-stain TEM of 1D4-eluted material demonstrates vesicles of the appropriate size, decorated with expected structures, with minimal contamination by nonvesicular structures. ***E***, Immunoblot of SV proteins in input fraction along with peptide and SDS eluates from rho1D4 beads, with approximate amount of total protein loaded per lane. ***F***, Immunoblot of peptide and SDS eluates probed with anti-mouse secondary mAb demonstrating the relative absence of eluted mAb in peptide eluates.

Although the anti-syt1 and anti-SV2 mAbs are highly effective for SV immunoprecipitation (Fig. 1), they do not readily allow for elution of native vesicles for downstream applications. The 9-mer 1D4 peptide sequence (TETSQVAPA) has been commercialized as a C-terminal affinity tag, as this peptide gently elutes rho1D4 mAb–bound targets under native conditions (MacKenzie et al., 1984; Wong et al., 2009). We thus attempted elution of rho1D4 bead-bound SVs with the 1D4 peptide (Fig. 3*A*) and found that a single incubation with the 1D4 peptide released ∼25% of the bead-bound protein, including ∼50% of the bead-bound synaptophysin (Fig. 3*B*). This eluate, which typically contained ∼25 ng/µl protein (Fig. 3*B*, Table 1), represents an enrichment of vesicular protein by several hundred-fold (Table 1). Examination of this eluate by dynamic light scattering demonstrated a population of particles 30–60 nm in diameter (Fig. 3*C*). Negative-stain TEM of these samples likewise demonstrated a population of vesicular structures 30–60 nm in diameter, studded with ∼8 nm tall structures that may represent the V1 sector of the V-ATPase (Fig. 3*D*).

**Table 1.** Purity and yield of SVs isolated by rho1D4-IP

Fraction	Volume, ul	[Protein], ng/ul	[Synaptophysin], normalized	Enrichment factor	Percentage of input synaptophysin	Preparation time, minutes
Input (supernatant from one-half mouse brain)	1900	2500 ± 300	1	1	100	30
rho1D4 bead-bound material	∼60	95 ± 11	7.7 ± 0.7	180 ± 30	∼20%	60
1D4 peptide eluate	∼60	25 ± 7.5	3.3 ± 0.6	330 ± 120	∼10%	90

Quantification of input protein was achieved by BCA assay, and quantification of SV yields was achieved as described in Materials and Methods. Data are shown as mean ± SEM for three immunoprecipitations using two mouse brains and three batches of rho1D4 beads. In contrast, SVs prepared using classical methods usually achieve synaptophysin enrichment factors of 20–30 when adjusted for the type of input fraction used here (Ahmed et al., 2013), whereas the SV-tag approach achieves an enrichment factor of 10–15 for synaptobrevin (Chantranupong et al., 2020).

**Figure 4.**
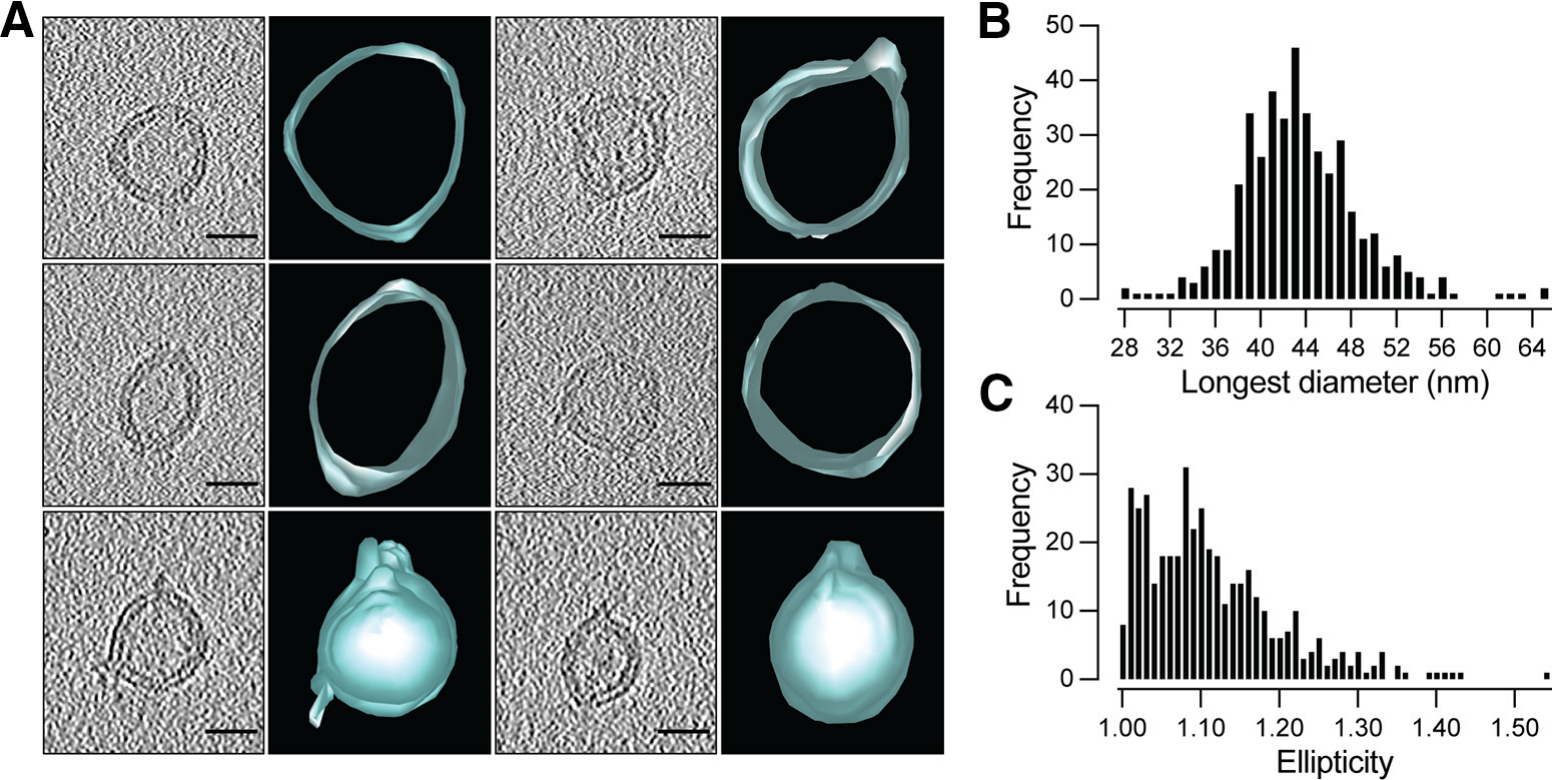
Cryo-electron tomography of synaptic vesicles obtained by rho1D4-IP. ***A***, Varied morphology of SVs as seen by cryo-electron microscopy and their corresponding three-dimensional reconstructions. Scale bar, 25 nm. ***B***, Frequency distribution of SV diameters measured along the longest axis (*n* = 421). ***C***, Frequency distribution of SV ellipticity defined as the ratio between the longest and orthogonal diameters of each vesicle.

We characterized peptide-eluted SV samples, which to our knowledge represent the most intact preparations of free SVs reported to date, with cryo-electron tomography (Fig. 4). We observed a striking variety of SV morphologies in our preparation, including oblong or angulated shapes, in addition to the expected spheres. Measurement of the longest and orthogonal diameters of each vesicle (Fig. 4*B*,*C*) yielded an average longest diameter of 43.4 nm, an average orthogonal diameter of 39.1 nm, and an average ellipticity of 1.11 (Fig. 4*C*). To our knowledge, this is the first characterization of nonspherical morphology in isolated SVs, as previous cryo-EM studies of purified SVs assumed a spherical shape in their morphologic characterization (Takamori et al., 2006). However, we note that studies of SVs *in situ* have identified nonspherical SVs for decades (Korneliussen, 1972; Rastad, 1981; Tao et al., 2018), although the causes of nonspherical SV morphology remain unknown. Even with the relatively lower contrast provided by cryo-EM without negative staining, electron densities on the outer surface of the SV membranes were observed (Fig. 4*A*).

**Figure 5.**
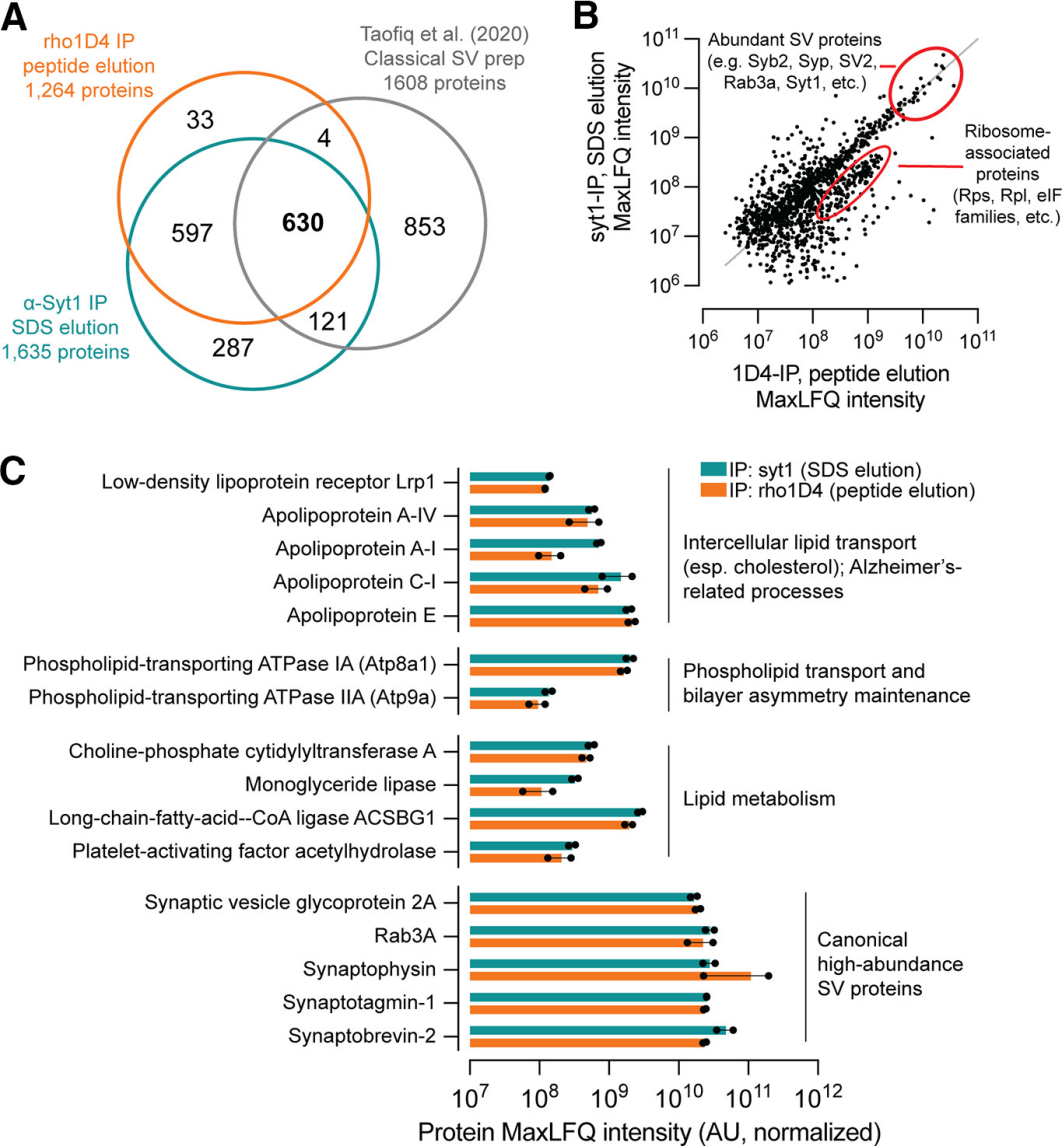
Synaptic vesicles copurify with proteins involved in lipid homeostasis, including ApoE and the lipoprotein receptor Lrp1. Vesicles were prepared by immunoprecipitation and elution with the 1D4 peptide (rho1D4 mAb beads) or SDS (α-syt1 mAb beads) and subjected to Orbitrap LC-MS (*n* = 2 biological replicates per purification method). ***A***, Comparison of protein identifications among syt1-IP, rho1D4-IP with peptide elution, and SVs prepared by classical methods (Taoufiq et al., 2020). ***B***, Scatter plot demonstrating robust correlation of intensity values for SVs purified by syt1-IP versus rho1D4-IP with peptide elution. All proteins with quantifiable intensity in at least one replicate were included. A small cluster of proteins enriched in Rho1D4-IP with peptide elution consists of ribosomal proteins that copurify with this method. ***C***, Proteins with well-established roles in lipid transport and metabolism were manually identified from the top 500 most intense proteins in each condition. Closed dots represent values from individual replicates. ApoE predominated among the detected apolipoproteins and was among the top 80 most intense proteins in all replicates, regardless of purification method. Atp9a and Atp8a1 are phospholipid flippases that maintain physiologic distributions of phosphatidylserine. Pcyt1a regulates phosphatidylcholine abundance via synthesis of phosphocholine, a rate-limiting step in phosphatidylcholine synthesis. Monoglyceride lipase cleaves monoacylglycerol molecules and plays key roles in endocannabinoid signaling. The fatty-acid-CoA ligase Acsbg1 activates long- and very-long-chain fatty acids with coenzyme A for downstream metabolic processing. Pla2g7 is a phospholipase A2 isoform important for immune function and breakdown of oxidized lipids. Abundance values for canonical high-abundance SV proteins are given for comparison. Source data used to generate this figure are shown in Extended Data Figure 5-1, and a comparison of proteins detected exclusively by classical approaches (Taoufiq et al., 2020) or by immunopurification (this study) is shown in Extended Data Figure 5-2.

We next performed proteomics experiments with deeper coverage enabled by high resolution and accurate mass measurements using an Orbitrap mass spectrometer and optimized capillary chromatography (Shishkova et al., 2018) to compare syt1 immunoprecipitates with vesicles obtained by rho1D4-IP and peptide elution (Fig. 5). These experiments demonstrated the high purity of both preparations and yielded deep coverage of the SV proteome, including vesicular acetylcholine and monoamine transporters (Extended Data Fig. 5-1). In comparison with a recent proteomic analysis of classically isolated SVs (Taoufiq et al., 2020), our results yielded similar numbers of protein identifications without the use of off-line prefractionation before nLC-MS/MS analysis (Fig. 5*A*), representing several hours of additional time savings. Importantly, our results demonstrated significant overlap with those of Taoufiq et al. (2020), identifying a group of 630 proteins detected using classical methods and the two separate immunoprecipitation methods described here. The proteins observed by Taoufiq et al. (2020), which were not observed in syt1 or rho1D4 immunoprecipitates, included many involved in vesicular transport, endocytosis, mitochondrial metabolism, and protein phosphorylation, consistent with techniques based on particle size fractionation rather than molecular features (Extended Data Fig. 5-2).

10.1523/JNEUROSCI.2521-21.2022.f5-1 Figure 5-1 This table contains the results of two independent mass spectrometry experiments processed via FragPipe and described in Figure 5. This table accompanies this article as a Microsoft Excel file with further instructions for its use noted on the first sheet. Download Figure 5-1, XLSX file.

10.1523/JNEUROSCI.2521-21.2022.f5-2 Figure 5-2 This table contains a list of proteins observed in the classical SV preparation of Taoufiq et al. (2020) not observed in our SV preparations, as well as a list of proteins observed in our preparations but not in Taoufiq et al. (2020). Download Figure 5-2, XLSX file.

Comparison of material obtained by syt1-IP and rho1D4-IP with peptide elution demonstrated a robust correlation between intensity scores of proteins detected in each sample (Fig. 5*B*), indicating that each IP method isolates a similar population of SVs. Even with peptide elution, Rho1D4-IP vesicles did contain some residual contamination, including a small cluster of ribosomal subunits (Rps, Rpl proteins) and initiation factor subunits (eIF proteins) enriched in these samples (Fig. 5*B*). Abundant contaminants in the classical SV preparation of Taoufiq et al. (2020), including glutamine synthetase and GAPDH, were observed in low abundance or were undetectable in our preparations (Extended Data Figs. 2-1, 5-1, 5-2). Among proteins observed in our IP samples but not in classical preparations, RNA-processing proteins predominated, suggesting that both rho1D4 and α-syt1 IP procedures may isolate small amounts of RNA and RNA-associated proteins (Extended Data Fig. 5-2). We note that ribosomal subunits have also been detected in classical SV preparations (Taoufiq et al., 2020) as well as in axonal proteomes (Chuang et al., 2018; Hobson et al., 2022), although the connection between ribosomal proteins and axonal membrane trafficking is not well defined.

We focused our annotation of these data on proteins that may modulate the lipid composition of nerve terminals because much remains unknown about the processes underlying lipid metabolism and homeostasis at nerve terminals. Most notably, we detected the lipoprotein apolipoprotein E (ApoE), along with several other apolipoproteins and the lipoprotein receptor Lrp1, in all SV preparations analyzed (Fig. 5*C*; Extended Data Figs. 2-1, 5-1). ApoE was among the top 80 most abundant proteins by MaxLFQ intensity in syt1 and rho1D4 peptide-eluted immunoprecipitates, and both ApoE and Lrp1 were previously detected in studies of classically isolated SVs (Takamori et al., 2006; Taoufiq et al., 2020). Several other proteins involved in lipid transport and metabolism were among the 500 most abundant proteins by MaxLFQ intensity (Fig. 5*C*), including the phospholipid-transporting ATPases Atp8a1 and Atp9a, fatty acyl-CoA synthetase ACSBG1, monoglyceride lipase, and the enzyme choline-phosphate cytidyltransferase A (Pcyt1a), which plays a key role in regulating phosphatidylcholine (PC) abundance. These proteins, with the exception of Pcyt1a, were likewise detected using classical SV isolation methods (Taoufiq et al., 2020). Because Pcyt1a undergoes reversible dissociation with membranes (Cornell and Northwood, 2000), it is possible that the overnight purification procedure used by Taoufiq et al. (2020), may have resulted in its dissociation from SV membranes. Alternatively, this discrepancy may have arisen during the computational assignment of detected peptides, as both our study and that of Taoufiq et al. (2020) also detected the closely related isoform Pcyt1b (Extended Data Figs. 2-1, 5-1). Finally, the top 500 most intense proteins also included the phospholipase A2 variant platelet-activating factor acetylhydrolase (Pla2g7), a secreted protein that metabolizes oxidized lipids and acts as an immunomodulatory protein (Tjoelker et al., 1995). Together, these findings indicate that SVs contain not only a host of enzymes involved in regulating membrane composition but also ApoE and Lrp1, which allow for ApoE internalization and recycling. These results, which are consistent with studies demonstrating the accumulation of ApoE and Lrp1 at nerve terminals (Bilousova et al., 2019), implicate the SV cycle in ApoE-mediated transport processes and suggest previously unappreciated roles for the SV cycle in the pathophysiology of neuroinflammation (Guttenplan et al., 2021) and age-related neurodegeneration (Bilousova et al., 2019).

## Discussion

This work details improved approaches for SV immunopurification (Matthew et al., 1981; Burger et al., 1989) and introduces rho1D4-IP as an accessible method for the purification and elution of SVs. Rho1D4-IP with peptide elution enables the rapid (<2 h) isolation of an exceptionally pure SV sample (Fig. 3, Table 1) without contamination from antibodies, exposure to proteases, or harsh physicochemical conditions. Future experiments may determine whether these vesicles undergo their native biochemical functions including membrane fusion and neurotransmitter loading. If native vesicles (Figs. 3–5) are not needed, techniques involving harsh elution from immunobeads provide a favorable compromise of purity for yield, as this material is still at least fivefold more enriched for SV proteins than SVs prepared classically (∼20–30-fold enrichment, Ahmed et al., 2013) or by using the SV-tag approach (∼10–15-fold enrichment, Chantranupong et al., 2020; Table 1). Although it does not enable molecular specification of target vesicle populations like SV-tag, rho1D4-IP represents a promising example of antibody repurposing and enables wide access to a new standard in purity and convenience for purifying SVs. The molecular interactions underlying the avidity of rho1D4 mAb–coated beads for SVs remain unclear but likely involve low-affinity binding between rho1D4 mAb and SV proteins along with polyvalency effects from multiple binding sites on both the bead surface and on each SV.

We emphasize the suitability of these approaches for studies with transgenic mice; a single P10–P20 mouse brain (0.3–0.4 g) comfortably provides enough material for two IP experiments (3 mg Ab-Dynabeads each) including protein and neurotransmitter analyses (Fig. 1). mAbs against syt1 and SV2 also provide a highly pure SV sample (Figs. 1, 2, 5, Extended Data Figs. 2-1, 5-1), although they do not presently allow for native vesicle elution. Importantly, hybridomas for both the α-syt1 and α-SV2 mAbs used here are available from the Developmental Studies Hybridoma Bank (https://dshb.biology.uiowa.edu/), which should enable their production in the required quantities by independent investigators.

Our proteomics analyses (Fig. 2, Fig. 5, Extended Data Figs. 2-1, 5-1) identified several proteins with previously unestablished roles in SV lipid metabolism that may regulate numerous elements of SV function. The abundance of the phosphatidylserine (PS) flippase Atp8a1 (Hiraizumi et al., 2019) suggests that SVs actively maintain the appropriate polarity of PS, and the presence of the PC biosynthetic enzyme Pcyt1a suggests a regulatory mechanism to maintain the PC content of SV membranes (Wang et al., 2005). The conical shape of PS suggests that Atp8a1 may also drive the formation of highly curved SV membranes (Takada et al., 2018) alongside the proteins synapsin and synaptophysin (Park et al., 2021). SVs also contain substantial quantities of the enzyme long-chain-fatty-acid—CoA ligase ACSBG1 (Fig. 5, Extended Data Figs. 2-1, 5-1), also known as lipidosin, which enables an early step in the metabolism of long- and very-long-chain fatty acids (Min and Benzer, 1999; Pei et al., 2003; Sheng et al., 2009). In mammals, this protein is selectively expressed in brain and testis (Pei et al., 2003), and its abundance in SVs raises further questions about the roles of SVs in neuronal lipid metabolism. The presence of the phospholipase A2 variant platelet activating factor acetylhydrolase (Pla2g7), also known as lipoprotein-associated phospholipase A2, suggests a mechanism by which cycling of SVs might drive metabolism of oxidized lipids and modulate the local immunologic environment (Tjoelker et al., 1995; Tellis and Tselepis, 2009) or presynaptic Ca^2+^ (Hammond et al., 2016). We emphasize that any number of lipid-dependent processes, including membrane trafficking and endocannabinoid signaling, may be shaped by enzymes that regulate the distribution of fatty acids at nerve terminals. Selective ablation of these proteins in models of neurodevelopment, behavior, and synaptic transmission will help clarify their functional roles.

Our LC-MS results also raise provocative questions about the role of SV cycling in presynaptic lipid homeostasis and clearance of the Alzheimer's disease–associated protein product amyloid-β (Aβ). Presynaptic dysfunction is an early hallmark of Alzheimer's pathology (Terry et al., 1991; Hsia et al., 1999; Hark et al., 2021), but much remains unclear about the molecular interactions underlying this presynaptic vulnerability. Lipid transport systems likely play critical roles; ApoE, a major component of the lipoproteins that enable neuronal cholesterol uptake, is functionally associated with Alzheimer's pathology (Corder et al., 1993; Li et al., 2012) and the clearance of extracellular Aβ (Strittmatter et al., 1993; Verghese et al., 2013). Molecular studies suggest cholesterol itself may drive pathologic protein aggregation at nerve terminals (Habchi et al., 2018). Moreover, the promiscuous endocytic receptor Lrp1 internalizes not only cholesterol-rich ApoE lipoproteins but also Aβ (Deane et al., 2004). However, although ApoE has been shown to accumulate in nerve terminals (Bilousova et al., 2019), and the SV cycle has been implicated as a key point of vulnerability in the early stages of Alzheimer's pathology (Hark et al., 2021), a connection between ApoE and Aβ uptake and the SV cycle has not to our knowledge been established. Such a role is not unexpected; SVs are cholesterol-rich organelles (Takamori et al., 2006), and neurons normally rely on astrocyte-synthesized lipoproteins containing ApoE for cholesterol uptake (Vance et al., 1994; Mauch et al., 2001; Fünfschilling et al., 2007; Nieweg et al., 2009). Strikingly, our proteomics experiments on immunopurified SVs revealed both ApoE and Lrp1 in anti-syt1 and rho1D4 immunoprecipitates (Fig. 5, Extended Data Figs. 2-1, 5-1), implying that ApoE may traffic in nerve terminals *via* the SV cycle. We note that these proteins have been consistently detected in SVs regardless of purification method; α-syt1-immunoprecipitation and elution with SDS, rho1D4-immunoprecipitation and elution by peptide or SDS (Fig. 5), or classical methods (Takamori et al., 2006; Taoufiq et al., 2020). Given recent evidence that reactive astrocytes may secrete cytotoxic lipids via ApoE lipoproteins (Guttenplan et al., 2021), further studies may explore the links among presynaptic lipid signaling, lipoprotein trafficking, neuroinflammation, and the SV cycle.

We emphasize that the notion of a pure SV preparation is somewhat simplistic, as trafficking organelles in brain will have varying degrees of SV-like identity based on protein composition or physical characteristics. Most SV preparations likely includes small populations of early and late endosomes, lysosome-like vesicles, and organellar intermediates on a continuum from Golgi-derived SV precursor vesicles to mature SVs. For example, published SV proteomics studies (Chantranupong et al., 2020; Taoufiq et al., 2020) and the present study (Extended Data Figs. 2-1, 5-1) report the detection of lysosome-associated glyocoproteins (LAMP1/2/5) at relatively low abundance in SV samples. As such, the presence of ApoE and Lrp1 in this preparation does not imply that ApoE traffics preferentially though the SV cycle—neither ApoE nor Lrp1 is limited to nerve terminals (Uhlen et al., 2015)—but our findings do suggest that some cotrafficking of ApoE-Lrp1 vesicles and SVs may occur. Although ApoE undergoes endocytic recycling (Heeren et al., 2001) and can be internalized and trafficked from distal axons (Amaratunga et al., 1996), its precise itinerary following endocytosis in the axon has not yet been established. ApoE may thus accumulate in SVs, which represent a uniquely vulnerable compartment (Hark et al., 2021), via SV recycling or other endosomal delivery pathways. In any case, the present results suggest further exploration of the SV cycle as a direct link among neuronal activity, lipid homeostasis, and proteostasis at the nerve terminal. Finally, our results suggest that modulation of synaptic activity or presynaptic membrane-trafficking processes, in addition to presynaptic lipid metabolism per se, may hold potential for the prevention or treatment of presynaptic neuropathological processes.
