# Supplementary figures and images for: Rapid and Gentle Immunopurification of Brain Synaptic Vesicles
Source: J Neurosci. 2022 Apr 27;42(17):3512–22. doi: 10.1523/JNEUROSCI.2521-21.2022 (PMC9053850; doi:10.1523/JNEUROSCI.2521-21.2022)

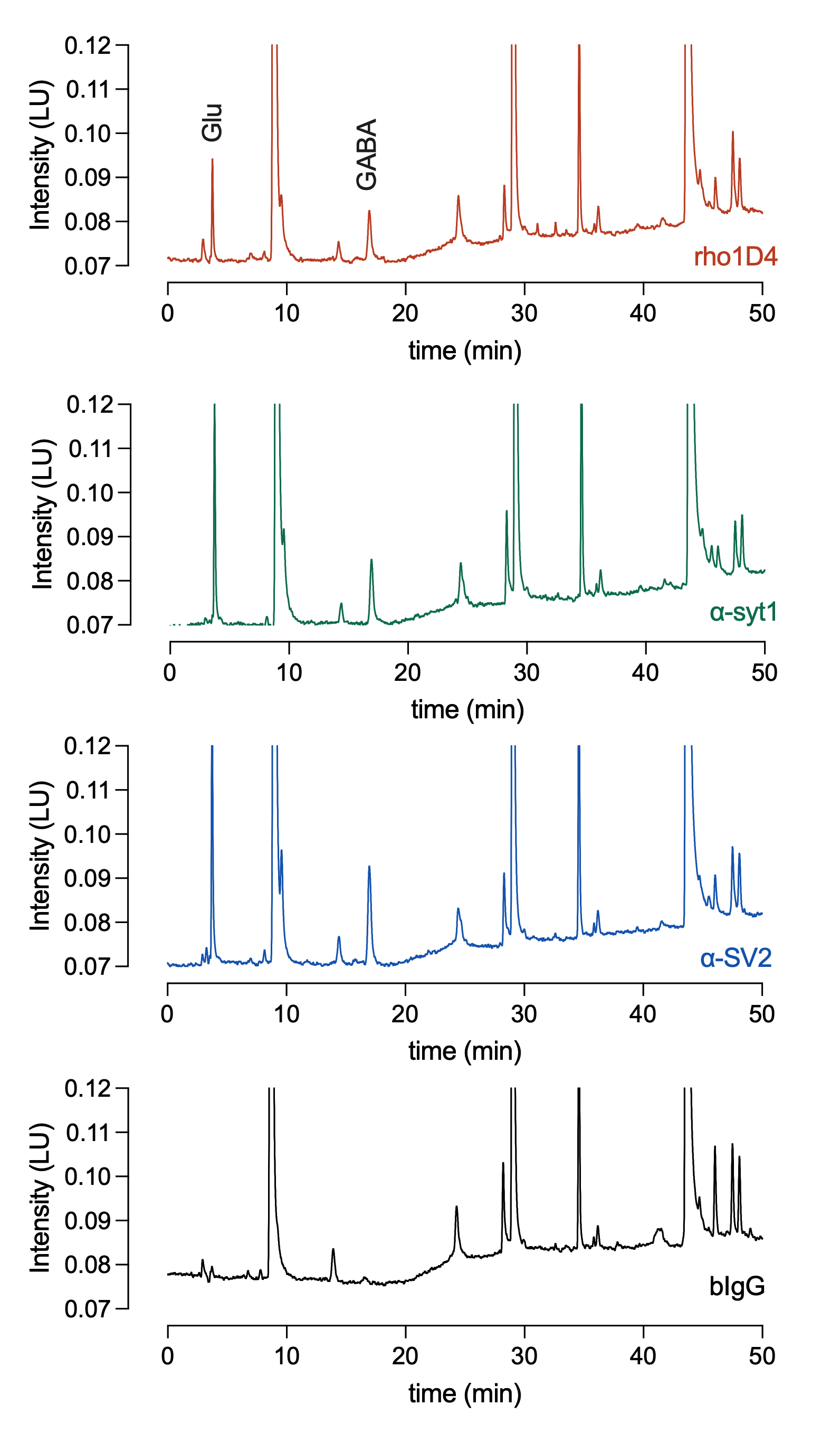

Supplement: Figure 1-1 — Chromatograms of derivatized polar amines eluted from immunoprecipitated SVs. Amines were detected by reverse-phase HPLC with fluorescence detection following elution from beads with 50% methanol and derivatization with NBD chloride. The elution positions of GABA and glutamate were established using standards. In accordance with other metabolomic studies of SVs (Burger et al., 1991; Chantranupong et al., 2020), GABA and glutamate were the predominant amines detected in this sample. Download Figure 1-1, TIF file. [file ns-JN-RM-2521-21-s04.tif]
